# Pediatric resident’s perception of night float system compared to 24 hours system, a prospective study

**DOI:** 10.1186/s12909-020-02474-x

**Published:** 2021-01-06

**Authors:** Fahad Alsohime, Hamad Alkhalaf, Hissah Almuzini, Malak Alyahya, Reema Allhidan, Ghadeer Assiry, Munirah AlSalman, Walaa Alshuaibi, Mohamad-Hani Temsah, Abdullah Alakeel, Ayman Aleyadhy

**Affiliations:** 1 College of Medicine, King Saud University, P.O. 231418, Riyadh, Riyadh 11321 Saudi Arabia; 2 Pediatric Intensive Care Unit, Pediatric Department, King Saud University Medical City, Riyadh, Saudi Arabia; 3 General Pediatrics and Complex Care, King Abdullah Specialized Children’s Hospital, King Saud bin Abdulaziz University for Health Sciences, Riyadh, Saudi Arabia; 4 Department of Pediatrics, Medical Generics Division, King Khalid University Hospital, College of Medicine, King Saud University, Riyadh, Saudi Arabia

**Keywords:** Duty hour, Post-graduate, Residency, Well-being, Work schedule tolerance, Night float

## Abstract

**Background:**

The study aims to evaluate the perceptions of pediatric residents under the night float (NF) on-call system and its impact on well-being, education, and patient safety compared with the traditional 24-h on-call system.

**Methods:**

The study is prospective in nature and conducted on two pediatric resident training centers who apply the NF on-call system as a pilot project. Senior residents (PGY-3 and PGY-4) enrolled in the two training centers were invited to participate before and 6 months after the implementation of the change in the on-call system. A self-administered online questionnaire was distributed. Responses were rated using a five-point Likert-type scale (1 = strongly disagree; 5 = strongly agree). The items covered three main domains, namely, residents’ well-being, ability to deliver healthcare, and medical education experience. Pre- and post-intervention scores were presented as means and compared by t-test for paired samples.

**Results:**

A total of 42 residents participated in the survey (female = 24; 57.1%). All participants were senior residents; 25 (59.6%) were third-year residents (PGY-3), whereas 17 (40.4%) were fourth-year residents (PGY-4). The participants reported that many aspects of the three domains were improved with the introduction of the NF system. The system was perceived to exert less adverse health effect on the residents (mean: 2.37 ± 1.01) compared with the 24-h on-call system (mean: 4.19 ± 0.60; *P* < 0.001). In addition, the NF system was perceived to lead to less exposure to personal harm and result in less negative impact on quality of care, better work efficiency, reduced potential for medical errors, more successful teaching, and less disruptions to other rotations compared with the 24 h on-call system (*P* < 0.001).

**Conclusion:**

The perception of senior residents toward the 24-h on-call system pertains to negative impacts on well-being, education, and patient safety compared with on-call systems with restrictive duty hours, such as the NF system, which is perceived to be less harmful, to exert positive impacts on the quality of delivered healthcare services, and more useful from pedagogic aspect.

## Background

For several decades, adjusting the duration of working hours and on-calls of residents-in-training has been a major concern worldwide. Several studies on the traditional 24-h on-call system reported that the system is associated with sleep deprivation and fatigue, which subsequently, result in increased medical errors and motor vehicle occlusions. Furthermore, others studies reported an increase in the incidence of burnout and suicide attempts among physicians. As a result, several countries have established a new system of work coverage called the night float (NF) shift system and abandoned the traditional 24-h on-call system [1–6].

The NF system is a work coverage system, where residents care for patients for 12–16 h during daytime or nighttime [7]. In other contexts, residents are divided into two alternating groups, where one group works at nighttime, whereas the other works during the day [8]. Alternatively, the traditional 24-h on-call system holds doctors responsible for receiving calls from the emergency department or a medical teaching unit for 24 h [9].

In 2003, the Accreditation Council for Graduate Medical Education (ACGME) in the United States limited working hours to 80 h per week and shifts to 30 h. Furthermore, it implemented restrictions that limited shifts for junior trainees to 16 h in 2011 [10]. In the same year, Quebec province in Canada restricted duty hours to no more than 16 h per shift [11]. In addition, Europe implemented restrictions on the duty hours of residents [12]. In 2017, Korea established the NF system for 6 months [8]. In Singapore, the NF system was applied to interns in an internal medicine department for a study [6].

Multiple studies have shown controversial results regarding the implementation of the NF system [2]. One of the drawbacks was reduced autonomy for cross-coverage interns [13] and decreased opportunity for residents in terms of education and learning because a majority of teaching and learning opportunities take place during the day [14]. The emergence of a shift–work mentality among residents was noted as a consequence of increased frequency of sign-overs, that is, the shift time became stricter, and the weak integration between daytime and NF teams [15].

Saudi Arabia adopted the traditional 24-h on-call system, and many residents previously worked for more than 24 h per day with unlimited number of on-calls per month depending on the hospital. In 2014, the Saudi Commission for Health Specialties (SCFHS) introduced new regulations for duty hours, where PGY-1 and PGY-2 (junior residents) will respond to a maximum of 7 on-calls per month, whereas PGY-3 and PGY-4 (senior residents) will attend to a maximum of 6 on-calls per month. Additionally, the average duration of an on-call should remain within 24 h, and residents should be off-duty maximally by afternoon of the following day [1].

Against this background, the current study evaluated the experience of pediatric senior residents (PGY-3 and PGY-4) with the NF system before and after its implementation as a pilot project in two pediatric residency training centers in Saudi Arabia compared with the traditional 24-h on-call system in terms of effects on health, education, and patient care and safety.

## Methods

The study is prospective in nature and was conducted on two pediatric residency training centers in Riyadh, Saudi Arabia from October 2018 to May 2019. Both training centers have a total of 86 residents (34 and 52 residents in each center), including 64 senior residents (25 and 39 senior residents in each center).

Both centers agreed to pilot the NF on-call system for all 64 senior residents (PGY-3 = 38 residents and PGY-4 = 26 residents) as a project that targets academic quality improvement. Approval was given by the pediatric scientific council at the SCFHS and senior residents from both centers.

The project involved changing the residents’ on-call shift from the traditional 24-h on-call system, where they previously received a maximum of six 24-h on-calls per month, to the NF schedule with 16-h night shifts plus a 1-h overlap at shift change for effective handover. The NF shift includes weekdays and weekends. Each senior resident will work for 3–4 consecutive night shifts from 4 pm to 8 am every 7 days without required daytime academic or clinical activities.

Before implementing the modality of the on-call system, a validated, evidence-based questionnaire was administered to assess the perceptions of residents regarding the implications of the duty-hour reform with permission from Fabreau et al. [16].

Six months after the introduction of the NF on-call system, the same questionnaire was distributed to the senior residents who participated in the new system (Appendix A). The questionnaire is composed of items were rated using a five-point Likert-type scale (1 = strongly disagree, 2 = disagree, 3 = neither agree nor disagree, 4 = agree, and 5 = strongly agree) to measure the degree to which participants agreed or disagreed with the statements. The items are related to three main dimensions, namely, well-being of senior residents (16 items), ability to provide quality healthcare (17 items), and experience in medical education (16 items).

The primary outcome was the change in the perception of senior pediatric residents toward their well-being, ability to deliver quality healthcare, and medical education experience at pre- and post-intervention as measured by the questionnaire.

The study received ethical approval from the Institutional Review Board (IRB) of King Saud University. Residents were informed that participation was voluntary and assured about the anonymity and confidentiality of their responses. Written informed consent was obtained from the participants before enrollment in the survey.

### Statistical data analysis

Means and standard deviations were used to describe the continuous variables, whereas the categorically measured variables were described using frequencies and percentages. The *compute* command in the analysis program was used to estimate the mean score for each domain using the sub-items after reverse coding negatively worded statements to align their direction with the main sub-construct magnitude, i.e., level of agreement. The paired sample t-test was used to assess the statistical significance of the mean indicators of physician satisfaction with the two abovementioned on-call systems. SPSS IBM Version20 was used for data analysis. A *P*-value of 0.050 was considered statistically significant.

## Results

### Participants

A total of 42 senior residents out of 64 (65%) responded to the survey. The response rate throughout the process remained at 65%. The final sample comprised 24 (57.1%) female residents; 25 (59.6%) and 17 (40.4%) were in the third (PGY-3) and fourth (PGY-4) years of training, as shown in Table 1.

**Table 1:** Demographic and professional characteristics of the participants (*N* = 42)

	Frequency	Percentage
**Sex**		
Male	18	42.9
Female	24	57.1
Residency Level		
PGY-3	25	59.6
PGY-4	17	40.4

PGY-3: Level-3 resident; PGY-4: Level-4 resident

### Perceptions of the impact of the NF system

#### Well-being

Findings indicated that the residents perceived significantly more negative impacts of the 24-h on-call system on their general well-being compared with the NF system (general effects on health, restriction on physical activities, impairment of the circadian rhythm, overall fatigue, and episodes of physical illness, as well as increased need to consume stimulants, such as cola and coffee) (*P* < 0.001). The NF system was significantly associated with enhanced energy levels compared with the 24-h on-call system (*P* = 0.041).

Moreover, the perception of potential for harm to the self for the two on-call systems was measured using two indicators, namely, safety during driving home after the on-call and workplace potential harm, such as needle-stick injuries. Analysis revealed that residents perceived more safety toward the two indicators with the NF system versus the 24-h on-call system.

In terms of resilience with trading on-call shifts among residents, the results were not significantly different between the two on-call systems (*P* = 0.830). However, the residents reported that the 24-h on-call system led to significantly less permissive (access) to free time to accomplish errands, was less family friendly, and more restrictive in terms of time allotment for research than the NF system (Table 2).

**Table 2:** Changes in perceptions regarding the impact of the 24-h on-call system on three dimensions (N = 42)

	Mean (SD; Likert rating)			
	24-h on-call system	Night float system	t/df = 41	*P*-value
Well-being				
*Promotes general wellness*				
Adversely affects health	4.48 (0.92)	2.45 (1.27)	8.53	< 0.001
Restricts participation in physical activities	4.67 (0.69)	2.38 (1.23)	11.45	< 0.001
Impairs the ability to adapt to changes in the circadian rhythm	4.33 (0.95)	2.55 (1.33)	7.85	0.003
Contributes to overall sleep debt	4.57 (0.70)	2.71 (1.42)	7.74	< 0.001
Contributes to overall fatigue levels	4.74 (0.59)	2.33 (1.26)	11.92	< 0.001
Contributes to frequent episodes of physical illness (e.g., colds)	3.90 (1.08)	2.14 (1.22)	7.9	< 0.001
Enhances overall energy levels	2.86 (1.52)	3.60 (1.23)	2.11	0.041
Contributes to the need to use stimulants, such as caffeine	4.40 (0.89)	2.86 (1.24)	7.5	< 0.001
Increases exposure to personal harm				
Impairs safety while driving home after an on-call	4.45 (0.86)	2.57 (1.19)	8.211	< 0.001
Increases potential for workplace harm, such as needle-stick injuries	4.10 (1.10)	2.21 (1.22)	9.3	< 0.001
Leads to conflicting role demands				
It is easy for me to trade on-call shifts with others.	3.07 (0.89)	3.02 (1.20)	0.22	0.83
Allows free time to accomplish non-work-related errands.	2.57 (1.33)	4.07 (1.20)	5.1	< 0.001
Provides opportunities for spending time with family	2.0 (1.15)	4.26 (1.08)	8.5	< 0.001
Restricts the time available for research	4.10 (0.98)	2.52 (1.27)	6.1	< 0.001
Promotes healthy relationships				
Allows healthy interpersonal relationships	2.55 (1.06)	4.17 (0.88)	6.86	< 0.001
Causes feelings of isolation Causes feelings of isolation at times	4.17 (0.85)	2.60 (1.33)	6.57	< 0.001
Ability to Deliver Quality Health Care Introduces potential for error				
In general, do you feel alert during procedures while on call?	2.90 (1.1)	4.14 (0.72)	5.83	< 0.001
Do you commit preventable medical errors?	3.64 (0.91)	2.55 (1.13)	5.23	< 0.001
Do you experience “near misses” related to poor patient care?	3.86 (0.75)	2.52 (1.02)	6.95	< 0.001
I am often extremely tired to provide safe patient care.	3.83 (1.06)	2.14 (1)	7.8	< 0.001
Promotes clinical skills expertise				
I miss important diagnoses.	3.83 (0.88)	2.17 (0.88)	9.62	< 0.001
I manage complex medical patients appropriately.	3.0 (1.08)	3.98 (0.87)	4.34	< 0.001
The content of my patient care handover is accurate.	3.19 (1.06)	4.11 (0.74)	4.79	< 0.001
I perform a thorough work-up of new admissions.	2.10 (0.96)	3.79 (1.18)	6.38	< 0.001
Promotes continuity of patient care I highlight important follow-up items during handover of patient care issues.	2.93 (1.24)	4.19 (0.77)	5.4	< 0.001
I maintain continuity of patient care.	2.71 (1.42)	4.57 (0.70)	7.74	< 0.001
I assume accountability for patients I admit.	3.57 (0.89)	4.12 (0.83)	3.41	< 0.001
Causes expenditure of emotional labor				
My interactions with other MTU team members are positive.	3.43 (0.99)	4.33 (0.57)	4.86	< 0.001
I communicate well with patients and their families.	3.55 (1.04)	4.21 (0.81)	3.42	< 0.001
I am sensitive to social issues pertaining to patient care (e.g., gender and culture)	3.19 (0.86)	3.29 (0.99)	0.703	0.486
**Promotes work efficiency**				
I can effectively multitask during busy work times.	2.67 (1.18)	4.0 (0.99)	5.99	< 0.001
I handover patient care issues in a time-efficient manner.	2.93 (1.24)	4.19 (0.77)	5.4	< 0.001
I respond to pagers in a timely fashion.	3.19 (1.21)	4.12 (0.86)	4.6	< 0.001
Medical Education Experience				
Promotes successful teaching				
I have enough time to teach junior residents and clerks.	2.19 (0.92)	3.83 (0.93)	7.84	< 0.001
I have enough energy to teach junior residents and clerks.	2.12 (0.92)	3.83 (1.06)	7.4	< 0.001
I am confident in my ability to teach procedural skills.	3.10 (1.14)	3.90 (0.93)	3.14	0.003
I am confident in my ability to teach regarding the management of unstable critically ill patients.	3.12 (1.10)	3.93 (0.95)	3.57	< 0.001
I am confident in my ability to teach the skills required for running a code.	2.93 (1.11)	3.60 (0.96)	2.9	0.006
Promotes medical skills proficiency				
I am confident in my ability to perform procedures.	3.48 (0.92)	4.14 (0.65)	3.72	< 0.001
I am confident in my ability to manage unstable critically ill patients.	3.45 (0.80)	3.90 (0.88)	2.3	0.026
I am confident in my ability to run a code.	3.19 (1.02)	3.71 (0.97)	2.67	0.011
Promotes successful learning				
I can acquire new knowledge on call.	3.33 (1.07)	4.12 (0.80)	3.76	0.001
I can retain new knowledge on call and apply it to patient care.	3.38 (1.08)	4.12 (0.86)	3.26	0.002
My overall education experience on call is satisfying.	2.83 (0.99)	3.95 (0.91)	5.46	< 0.001
I gain opportunities to learn procedures through simulation training.	3.36 (1.06)	3.86 (0.93)	2.51	0.016
Promotes staff physician supervision				
I have the opportunity to review cases with attending physicians.	2.95 (0.99)	3.79 (0.92)	4.14	< 0.001
My clinical skills (history and physical) are observed by an attending physician.	2.33 (1.0)	3.10 (1.16)	4.1	< 0.001
I receive feedback from attending physicians.	2.74 (1.06)	3.24 (1.19)	2.86	0.007
Causes disruptions in rotations				
My ambulatory care rotations are frequently interrupted due to MTU on-call duties.	3.95 (1.06)	2.38 (1.08)	5.55	< 0.001

With regard to the indicators of the resident’s relationships with others, analysis demonstrated that residents significantly felt more isolated under the 24-h on-call system reported significantly better social relationships under the NF system (*P* < 0.001).

#### Ability to deliver quality healthcare

Importantly, the perception of the impact of the on-call systems on the quality of delivered healthcare services by residents to patients was measured using four indicators. Data revealed that the residents perceived that they were significantly less alert during the 24-h on-call system compared with the NF system. Additionally, the residents perceived the 24-h on-call system as significantly associated with the density of preventable medical errors, more near-miss errors, and more fatigue, which influence the quality of care received by patients.

Analysis of the emotional burden on the residents indicated that they experienced significantly greater interaction and better communication with patients under the NF system compared with the 24-h on-call system. Conversely, the data demonstrated no statistical difference on sensitivity to social issues related to patient care and care planning, such as cultural and gender sensitivities, under the two on-call systems (*P* = 0.486). The indicators of work efficiency showed that the residents reported significantly less multitasking ability, less handover efficiency, and less ability to attend pager buzzes during the 24-h on-call system compared with the NF system (*P* < 0.001).

In terms of the impact of the two on-call systems on aspects of residents’ expertise, the residents perceived that they would significantly miss important diagnoses of their patients, be less able to manage complex medical issues, provide less accurate medical handovers, and feel less accountability for patient care under the 24-h on-call system (*P* < 0.001) compared with the NF system (Table 2).

### Medical education experience

Table 2 displays the residents’ responses on emotional burden, efficiency of work, education, skills, learning ability, supervision, interruptions during rotations, and post on-call.

Under the 24-h on-call system, the teaching of junior residents and clerks was significantly less time-permissive and more energy consuming compared with the NF system. In the 24-h on-call shifts, residents perceived that they were less confident to teach, especially during the management of unstable or emergency patients or during codes. The indicators of skillfulness suggested that residents felt less confident with performing medical procedures, managing critically ill patients, and conducting cardiopulmonary resuscitation under the 24-h on-call system than the NF system (*P* < 0.050 each).

With regard to learning, the data demonstrated that residents perceived less acquisition of knowledge, less usage of new knowledge, and less satisfaction with education or teaching sessions via simulation under the 24-h on-call system than the NF system (*P* < 0.050 each). Furthermore, the residents perceived the 24-h on-call system as significantly less helpful for reviewing cases with peers, less permitting of in-depth discussions of clinical skills, and leading to less feedback from attending seniors compared with their experience under the NF system (*P* < 0.050 each).

The residents perceived the 24-h on-call system as significantly more interruptive to ambulatory care rotations and associated with more post on-call call-backs and fatigue during weekends, which influence successive weekday work rotations compared with the NF system (*P* < 0.001 each).

#### Overall rating of the three domains

Analysis of the overall experience of the residents under the two on-call systems indicated that the residents perceived the NF system as having a positive impact on general wellness, more role resilient, and promoting healthier social and family relationships. In addition, the residents felt less exposed to harm and risk with better quality of patient care. Furthermore, the residents experienced more resilience with regard to the emotional integrity of patient care compared with the traditional 24-h on-call system.

Also, the residents felt they were working more efficiently with increased teaching ability and skillfulness, better learning, and more efficient supervision during the NF on-calls. Table 3 provides the means and standard deviations.

**Table 3:** Changes in perception regarding the impact of the on-call systems on three dimensions (N = 42)

	Mean SD; Likert rating			
	24-h on-call system	Night float system	t/df = 41	*P*-value
Well-being				
Promotes general wellness	4.19 (0.60)	2.37 (1.01)	11.32	< 0.001
Increases exposure to personal harm	4.27 (0.8)	2.39 (1.07)	10.17	< 0.001
Causes conflicting role demands	2.74 (0.59)	3.71 (0.77)	5.63	< 0.001
Promotes healthy relationships	2.19 (0.76)	3.79 (0.99)	7.4	< 0.001
Causes feelings of isolation	4.17 (0.85)	2.60 (1.33)	6.57	< 0.001
Ability to deliver quality healthcare				
Increases potential for error	3.61 (0.67)	2.27 (0.79)	7.8	< 0.001
Promotes clinical skills expertise	2.98 (0.75)	4.01 (0.61)	6.6	< 0.001
Promotes continuity of patient care	3.57 (0.89)	4.12 (0.83)	3.41	< 0.001
Causes expenditure of emotional labor	3.39 (0.74)	3.94 (0.61)	4.14	< 0.001
Promotes work efficiency	2.93 (1.01)	4.1 (0.75)	6.19	< 0.001
Medical education experience				
Promotes successful teaching	2.69 (0.82)	3.82 (0.85)	5.56	< 0.001
Promotes medical skills proficiency	3.37 (0.75)	3.92 (0.71)	3.16	0.003
Promotes successful learning	3.23 (0.83)	4.01 (0.79)	4.34	< 0.001
Promotes staff physician supervision	2.67 (0.80)	3.37 (0.92)	4.38	< 0.001
Causes disruptions in rotations	4.10 (0.75)	2.40 (0.98)	7.36	< 0.001

### Discussion and literature review

The study provides insight regarding the experience of senior residents under the newly implemented NF system in Saudi Arabia. Specifically, the study compares the perceptions of senior pediatric residents (PGY3 and PGY4) and their experiences under the NF system versus the traditional 24-h on-call system. Furthermore, the study examines the impact of the two settings in two academic pediatric tertiary care centers based on three important domains. The findings indicate that the NF system improves the overall on-call experience in terms of wellness, education, and patient care and management as perceived by the senior residents enrolled in the study. This result is similar to that of another study conducted in Korea [8]. A similar study conducted in Saudi Arabia found the same results: the 24-h on-call system exerted a negative outcome on the health and education of residents and safety of patients [1].

The survey mainly aimed to examine the effect of the two systems on the well-being of senior residents. The results indicated that senior residents perceived a positive impact on well-being under the NF system compared with the 24-h on-call system according to various indicators, such as general health, restriction of physical activities, overall fatigue, and episodes of physical illness. These results are similar to those of several studies, which illustrated the association between the traditional 24-h on-call system and loss of sleep and fatigue, which negatively impacts physical function and memory because extensive duty hours may cause burnout and thus lead to depression, substance abuse, and suicide attempts [2, 3, 5, 17]. This association was attributed to the impairment of the circadian rhythm [2]. Conversely, the NF system enabled residents to work within scheduled on-call hours, which led to an overall balanced life. This finding is evident in our data, which reported that residents experienced better interpersonal relations with more time to spend with family and less feelings of isolation compared with the 24-h on-call system.

Another aspect investigated was the quality of healthcare delivered to patients. The study noted improvement in patient care and communication perceived by the residents, which is similar to another study on the implementation of the NF system in another hospital [18]. In a study in Singapore on interns, a decreased in the incidence of medical errors was noted [6]. Our study could not reach this conclusion as the analysis focused only on the perception of senior residents in terms of making medical errors, which occurred more under the 24-h on-call system, without specific data. However, other studies found negative outcomes for patients care associated with the NF implementation, especially in the surgical and critical care areas [19]. Handover in the NF system was perceived as more efficient, a finding that was previously associated with improved patient care [20, 21]. The NF system mitigated this risk as daytime senior residents handed over directly to the night-shift senior residents and vice versa compared with the traditional 24-h on-call system, where a daily handover is conducted to different residents between shifts and between caring teams, which may be associated with high rates of medical errors and adverse events [22].

Certain doubts emerged that the NF system may reduce the quality of the education of residents [8] as well as opportunities for education due to decreased interactions and conferences under the NF system [23]. Interestingly, the study found that the perception of senior residents toward educational experiences and opportunities for learning procedures were in favor of the NF system. This finding may be attributed to the fact that residents are rested and more alert and energized during their shift. Hence, they are more open to receive learning from supervisors and perform procedures and more willing to teach juniors. Furthermore, the night shift employs less residents, which leads to increased one-to-one sessions with the attending physician for the discussion of cases and feedback as well as less competition on performing procedures. Such opportunities can be missed during the 24-h on-call system due to fatigue and exhaustion, which will render residents less interested in learning new knowledge or skills.

Bolster et al. [24] conducted an updated systematic review and demonstrated a potential negative impact on the education of residents. However, the authors also provided conflicting results, where one study indicated improvement in the learning of trainees [25], whereas another study found that the NF system did not alter the quality of education [6]. This result differs from that of the current study because the senior residents reported a better overall education. This difference may be due to the fact that the current study involved senior residents with more experience and, thus, better knowledge and skills.

One possible explanation for the conflicting results is the existence of different formats of the NF system, which may influence its educational value, and the variation in the evaluated clinical education components across studies.

On the contrary, another cluster-randomized trial by Desai et al. [26] compared between two duty-hour policies as implemented by internal medicine residency programs. The first policy pertains to those of the standard 2011 ACGME duty hours, where duty-hour periods should not exceed 24 h with an additional 4 h permitted for transitions in care. The second refers to flexible duty-hour policies with no limits on shift length or mandatory time-off between shifts. Although the actual number of work hours was not measured within the flexible duty-hour system, the authors found no significant between-group difference in the primary outcomes (trainee satisfaction) with balance between education and work. This finding signifies that restricted duty hours should be well structured and should be re-evaluated to achieve objectives in terms of avoiding the negative impact on the wellness and education of residents as well as patient care because the rules of the training programs aim to protect patients and trainees at the same time .

The study is limited in that it is a survey based on a self-administered questionnaire, which is subject to recall bias. Additionally, it is a perception-based survey without objective data or measures of patient outcomes. However, the study considers that the perceived impact of the NF system on the well-being and education of the senior residents and patient safety is an important factor that influences these aspects.

## Conclusion

The perception of senior residents toward the 24-h on-call system indicated negative impacts on their well-being and education and patient safety compared with the NF system. The restricted duty hours were perceived as less harmful to well-being and more useful from the pedagogic aspect and to exert positive impacts on the quality of delivered healthcare services. Pediatric residency training programs in Saudi Arabia should consider a further evaluation of the reform in duty hours for residents and explore new on-call models to improve resident well-being and training and enhance patient care. Examining objective outcomes, such as morbidity and mortality among patients or scores of different in-training assessment tools for trainees should be considered in the evaluation process.

## Data Availability

The datasets generated and/or analyzed during the current study are not publicly available but are available from the corresponding author on reasonable request.

## Abbreviations

NF: Night Float
ACGME: Accreditation Council for Graduate Medical Education
PGY-3: Level-3 resident
PGY-4: Level-4 resident
